# The role of 18F-FDG-PET/CT in the preoperative staging and posttherapy follow up of gastriccancer:Comparison with spiral CT

**DOI:** 10.1186/1477-7819-9-75

**Published:** 2011-07-14

**Authors:** Elgin Ozkan, Mine Araz, Cigdem Soydal, Ozlem N Kucuk

**Affiliations:** 1 From the Department of Nuclear Medicine, Ankara University, Medical Faculty, Ankara, Turkey

**Keywords:** Gastric cancer, FDG-PET/CT, spiral CT

## Abstract

**Background:**

The aim of this study was to investigate the role of F-18 fluoro-deoxy-glucose (FDG) positron emission tomography and computed tomography (PET/CT) in the preoperative and posttherapy restaging of gastric cancer and to compare with spiral computerized tomography (CT).

**Method:**

A total of 42 PET/CT scans of 36 gastric cancer patients (28M, 8F; mean age: 56,0 ± 15) were included in the study. A retrospective analysis of the PET/CT results of the patients were compared with concurrent CT results. Confirmation was made by clinical course and serial imaging studies in the follow up. The compatibility ratios were calculated and the accuracy of the PET/CT was assessed. Agreement between PET/CT and concurrent CT was calculated using kappa statistics.

**Results:**

Patients were separated into 3 groups: the patients who were referred to our clinic for preoperative staging (4 patients), for posttherapy evaluation (24 patients) and for the suspicion of local recurrence and/or metastasis exploration after a disease free period (8 patients). Groups 1 and 3 included a small number of patients so they were omitted from the statistical analysis. Focusing on Goup 2, the overall concordance rate was 50% (12 patients). Region based analysis showed the rates of concordance for local recurrence, local lymph node metastasis and distant metastasis were 91% (Kappa: 0.70), 95% (Kappa:0.86) and 50% (Kappa:0.26) respectively. Distant metastases were also investigated in detail and the two techniques showed a concordance of 91% (Kappa: 0.75) for liver, 79%(Kappa:0.31) for distant lymph node, 79% (0.42) for lung, 87%(Kappa:0.33) for bone and 95% for intestinal wall metastasis.

**Conclusion:**

PET/CT is a complementary imaging method which can be successfully used in both preoperative and posttherapy evaluation of gastric cancer.

## Background

Gastric cancer is the fourth most frequent type of cancer and 934.000 new cases arise each year worldwide [1]. Japan, China, East Europe and Latin America are reported as areas of high incidence of gastric cancer. The survival rates are generally very low because the patients usually have a high stage disease at diagnosis [2].

The only curative therapy for gastric cancer is the resection of both the tumor and the regional lymph nodes at the early stage of the disease. The evaluation of tumor resectability, local lymph node and regional solid organ and distant metastasis in the preoperative stage plays a crucial role in terms of planning a true surgery or avoiding unnecessary surgical interventions in high stage patients. Computed Tomography (CT) is frequently used for preoperative staging in gastric cancer patients. Endoscopic ultrasonography (USG) is known to be the most reliable method in the preoperative T staging of the disease [3,4]. However, the high technology multislice CT systems are reported to give results as accurate as endoscopic USG [5,6]. For nodal staging and evaluation of distant metastasis, spiral CT is also currently the method of choice in the preoperative stage [7]

In gastric cancer patients, detecting the recurrences is hard in the posttherapy follow up period. An elevation in the tumor markers like carcinoembryonic antigen (CEA) and Ca19-9 may help but there is still a need of a reliable method for the localization of recurrence. CT is the preferred method for this aim. However it is reported that CT has a limited value in the evaluation of the postoperative changes [7]. To determine the therapy response, the volumetric changes on CT is similarly used in the routine procedure. But especially in the detecion of the response in the primary tumor, these changes may not always be realized accurately [7]. When it comes to the detection of the solid organ and especially distant metastasis, although CT is currently used, there are some reports showing that as a whole body imaging method, Positron Emission Tomography (PET) is superior to anatomic imaging tools. But the role of 18F-Fluorodeoxyglucose (FDG)-PET/CT in the diagnosis of distant lymph node, bone or lung metastasis is uncertain [8].

18F-FDG-PET is a functional imaging method detecting the metabolically active tumor. It is well known that the primary energy source for cancer cells is glucose. Active tumor cells have an uncontrolled growth and division and therefore their metabolism mostly depend on anaerobic respiration which requires a greater amount of glucose consumption compared to the healthy tissues. FDG enters into the cell and is phosphorylated by hexokinase activity but can no longer be metabolized. Therefore it is trapped in the cell. Highly active malignant cells concentrate more FDG than normal tissues which provides the functional imaging in cancer patients. Hybrid PET/CT systems provide fusion images combining functional and anatomic imaging together [9].

The aim of this study was to investigate the role of F-18 fluoro-deoxy-glucose (FDG) positron emission tomography and computed tomography (PET/CT) in the preoperative and posttherapy restaging of gastric cancer and to compare with conventional CT.

## Method

### Patient Group

In this retrospective analysis, we reviewed a total of 51 PET/CT reports of 44 primary gastric adenocarcinoma patients to whom PET/CT was performed in the preoperative stage or for posttherapy restaging between January 2007 and January 2010. We used the clinical follow up registery of our hospital in order to reach the reports of their conventional imaging examinations and other investigations. We couldn't get the results of the medical examinations of 8 of these patients (a total of 9 PET/CT scans) who were referred only for PET/CT scan to our centre. They were omitted from the study. As a result, 36 gastric cancer patients (28M, 8F; mean age:56,0 ± 15) and 42 PET/CT reports were included in the study. 5 patients have undergone at least 2 PET/CT scans. The concurrent thoracoabdominal CT results were compared with the PET/CT results. Also, some of them had undergone additional imaging examinations like USG, Magnetic resonance imaging (MRI) or bone scintigraphy; so they were also taken under consideration. Confirmation was made by clinical course and serial imaging tests.

The subjects were divided into three groups. 4/42 of the analysed scans were performed for preoperative staging (group 1), 30/42 of the for posttherapy restaging (Group 2) and 8/42 for recurrence or metastasis search because of tumor marker elevation in the disease free follow up period (Group 3). Groups 1, 2 and 3 included 4, 24 and 8 patients respectively.

### PET/CT and Spiral CT

All spiral thoracoabdominal CT examinations were performed with oral and i.v. contrast agents. PET/CT imaging was done using the GE Discovery ST- 8 slices scanner. PET scans were performed after 6 hours of fasting. Blood glucose levels were checked just before the procedure. Average 296-370 MBq (8-10 mCi) FDG were injected intravenously and images were obtained 1 hour later from the orbitomeatal line to the mid thigh. Low dose CT images were used for attenuation correction. Oral contrast agent was given to all patients during PET/CT imaging. A semiquantitative and visual analysis was made. Images were evaluated by two nuclear medicine specialists and a consensus was reached in order to avoid interobserver variability. A focal uptake with a SUV>2.5 was considered pathological.

### Statistical analysis

Because the number of patients included in Groups 1 and 3 were not meaningful enough to be analysed statistically, statistics focused on the second group of 24 patients.

The results of PET/CT and conventional CT studies were compared in terms of the recurrence in the primary gastric tumoral focus, abdominal lymph node metastasis and distant metastasis.

Then the aggrement between two imaging techniques were checked by calculation of Kappa statistics. The analyses were performed using the SPSS software, version 11.5.0 (SPSS Inc.,Chicago,Illinois,USA).

Then the concordance and discordance between these two imaging modalities were investigated by checking the reliability. As most of the patients included were in the restaging group, the gold Standard histopathological confirmation could not be possible. So, sensitivity, specificity, positive and negative predictive values and accuraccy could not be calculated

## Results

In 16/36 (44%) patients, the results of the PET/CT and CT studies showed completely concordant findings and no additional foci were detected by PET/CT. Of these 16 patients, 2/16 were in the first group, 12/16 in Group 2 and 2/16 in Group3.

In group 1, 2/4 patients (50%) had compatible PET/CT and CT results. In the other half of these patients PET/CT gave more accurate results than thoracoabdominal CT examinations.

In 1/2 of these patients, PET/CT showed increased metabolism in the abdominal lymph nodes in addition to the lesions also detected by CT. These foci were all confirmed histopathologically after surgery. In the other 1/2 patient, the millimetric lung nodules diagnosed by thorax CT were non-FDG avid. Although the possibility of metastasis cannot be totally eliminated in millimetric non-FDG avid nodules, because no changes developed in either size or characteristics of the nodule, lung metastasis was not clinically considered in this patient.

Group 2 included 24 patients. The number of patients who had compatible results of PET/CT and CT in group 2 (overall concordance) was 12/24 (%50). The accuracy of the PET/CT results in the discordant group (12/24) were confirmed clinically. 1/12 of them also had histopathological confirmation. The two imaging methods showed concordant findings in 22 patients (%91) for local recurrence and in 23 patients (95%) for local lymph node metastasis. The evaluation of distant metastasis showed a rate of 50% overall concordance. The subgroup analysis of distant metastases was also done. The regions of distant metastasis noted were liver, distant lymph nodes, lungs, bones and intestinal wall. The number of patients who had concordant findings for defining liver metastasis was 22 (91%) (Kappa:0.75), distant lymph node metastasis was 19 (79%) (Kappa:0.31), lung metastasis was 18 (75%) (Kappa:0.42), bone metastasis was 21 (87%) (Kappa:0.33) and intestinal wall was 23 (95%). Becasue there was only one patient who had intestinal wall involvement, Kappa could not be calculated (Table 1).

**Table 1:** The rates of concordance and discordance and calculated Kappa values for PET/CT and spiral CT.

	CONCORDANCE	DISCORDANCE	KAPPA
**LOCAL RECURRENCE**	22 (91%)	2 (9%)	0.7

**LOCAL LYMPH NODE METASTASIS**	23 (95%)	1 (5%)	0.86

**DISTANT METASTASIS**	12 (50%)	12 (50%)	0.26

***LIVER***	22 (91%)	2 (9%)	0.75

***DISTANT LYMPH NODE METASTASIS***	19 (79%)	5 (21%)	0.31

***LUNG***	19 (79%)	5 (21%)	0.42

***BONE***	21 (87%)	3 (13%)	0.33

***INTESTINAL WALL***	23 (95%)	1 (5%)	-

**OVERALL**	12 (50%)	12 (50%)	-

In Group 3 there were 8 patients referred upon detection of high tumor marker levels with a local recurrence or distant metastasis suspicion. In 2/8 patients (25%), the two imaging modalities showed completely the same lesions. In 6/8 (75%) patients however, it was clinically confirmed that the extra lesions shown by PET/CT were positive. There were no lesions detected by CT but not with PET/CT.

## Discussion

The most reliable noninvasive tool routinely used for the preoperative TNM staging of gastric cancers is spiral CT. But as an anatomic imaging method, CT is known to have a low sensitivity and spesificity at N staging of the disease[10]. Because it basically names the lymph nodes pathological if there is an increase in the size and this may fail if the change in size is due to inflammatory process [11]. So PET/CT has evolved as a promising metabolic imaging modality not only showing the morphology but also the pathological metabolic activity of the tumoral tissues. The studies comparing CT with PET alone in the preoperative lymph node staging of gastric cancer reported that PET is not superior to CT. This is related with the low resolution of PET and perigastric lymph nodes cannot be distinguished easily from the primary tumor. But combined PET scans with CT provides a precise localisation like it is in our study (95%) [12-14]. In 1/4 patients to whom PET/CT was performed in order to make staging in the preoperative stage, FDG uptake was observed in the gastric region and CT showed that this activity was belonging to pathological regional lymph nodes not reported as pathological on spiral CT. The superiority of PET/CT, a metabolic imaging tool, over CT in the preoperative N staging is marked in this case.

In the literature, although PET/CT does not have a role in T staging in the preoperative state, sensitivity and specificity of PET/CT in showing the primary tumor is reported as %58-94 and %78-%100 respectively [9]. In all patients in group 1, compatible to CT, the primary tumoral focus showed FDG uptake in our study. It is an important limitation that the number of patients is small especially in the first group, the number of studies in the literature investigating the role of PET in the diagnosis of distant metastasis preoperatively is also very small. In a series reported, sensitivity and spesificity of PET in the diagnosis of liver metastasis was 85% and 74% respectively, 67% and 88% in lung metastasis [15]. In our study, in 1/4 of the Group 1 patients had lung nodules on CT but these nodules didn't show FDG uptake and no pathological change in size or characteristics developed, so they were clinically accepted as non metastatic.

Group 2 included the 24/36 patients who have undergone PET/CT in the posttherapy follow up. A total of 30 PET/CT scans were performed to these patients. When these scans were analysed on the lesion basis, the overall compatibility of CT and PET/CT scans was 50%. The results were generally compatible for regional recurrences (91%, Kappa:0.7) or local lymph node metastasis (95%, Kappa:0.86) but the incompatibility was mainly due to distant metastasis (50% Kappa:0.26). Enough information could not be provided in the literature about the role of PET/CT in this group of patients. We think that our clinical experinces given in this report will contribute the literature.

In 2/24 patients with incompatible results (9%) for the evaluation of operated zone, local recurrence was clinically confirmed despite a normal spiral CT. In the literature no conventional imaging method is reported to have sensitivity or specificity good enough for a reliable evaluation [16]. FDG PET also has a low sensitivity in detecting local lymph nodes. PET/CT hybrid systems theoretically have a higher senstivity but further studies with large patient groups are needed in order to reveal the true best modality. While the two techniques were generally compatible in the detection of local lymph node metastasis, in 1/24 (5%) patients, local lymph nodes showed pathological FDG uptake although they were not apparent on CT.

When it comes to the distant metastasis, the results of the analysis for liver metastasis showed that in 2/24 (9%) patients there was a strong suspicion that the liver metastasis still existed after chemotherapy as dynamic MRI or hepatobiliary studies have suggested. However no FDG uptake was seen in these areas. In the clinical follow up, it was proved that this situation was related with the early metabolic response to therapy before anatomic response became evident. As demostrated in these cases, we concluded that PET/CT has an important role in determining the early metabolic response to therapy before anatomic response develops or that chronic non specific changes not certainly distinguishable from malignity can be clarified by PET/CT.

Although mostly compatible findings with CT were obtained, PET/CT showed additionally extra uptake in distant lymph nodes in 5 patients (21%). In these high stage patients who have undergone surgery and multiple combined chemo-radiotherapies, restaging with lymph node sampling was not clinically appropriate. Because the minimum SUVmax value measured in these additional lymph nodes was 4.1, they were clinically accepted metastatic. A cut-off SUVmax value for lymph nodes to accept as malignant in gastric cancer patients has not been reported yet. Kim and colleagues accepted the cut off SUVmax value as 2.5 in their study at which the role of PET/CT was investigated, and they found out the sensitivity of the technique 40% [12]. In our study, because there was no histopathological confirmation, statistical analysis was not possible, but because the SUVmax values we reported were much higher than their cases, the sensitivity of PET/CT in our study is probably higher.

In 5/24 of the patients (21%) in group 2, no FDG uptake was seen in the milimetric lung nodules detected by CT. No clinically evident metastasis was seen in the clinical follow up or increase in size was detected in the following control CT examinations.

In the evaluation of the skeletal metastasis, the rate of discordance was 13% (3/24 patients) although CT showed no bone lesions in 1/24 patients, PET/CT showed the diffuse bone metastasis throughout the body which was proved by bone scintigraphy and the patient was already clinically symptomatic. In 2/24 of the patients however, PET/CT failed to show the sclerotic lesions reported on spiral CT.

In 1/24 patient, the abdominal CT was normal but pathological uptake was seen in the intestinal wall at the splenic flexura and the rectum on PET/CT (SUVmax:8,3 and 14,0). The colonoscopic biopsy results confirmed that these foci were related to gastric adenocancer metastasis.

In 2/8 patients in group 3, compatible results were found. In these 2 patients because both PET/CT and diagnostic CT scans were reported as normal, patients were taken under routine follow up. In the other 5/7 patients, PET/CT has additionally showed multiple abdominal lymph nodes in 1 patient, local recurrence and multiple bone metastases in 1 patient. In 1/5 patient while a single focus of liver metastasis was reported on CT, PET/CT revealed multiple metastasis in the liver with SUVmax:8.9. In this case PET/CT was helpful in detecting the new metastatic foci which are metabolically active but not radiologically visible yet. This patient was taken under a chemotherapy programme again. Lesions in the lung in 1 patient and in the proximal jejunal segments of the intestine in another patient were not FDG avid. So they were accepted as non malignant and no therapy indication was discussed clinically.

As per the National Comprehensive Cancer Network (NCCN) guidelines published, the role of FDG PET in the preoperative staging of gastric cancer is said to be still uncertain but it is most useful in detecting advanced disease [14]. Our results corroborate this guideline. We found that PET/CT is complementary to conventional CT in detecting distant metastasis at high stage disease. In the preoperative period, PET is not yet accepted because it cannot give the exact T stage and N stage of the disease, but in our study hybrid PET/CT was confirmed to be as successful as spiral CT in N staging, although T staging was of course not possible. So hybrid PET/CT systems may much probably be superior to PET alone as they can provide extra information of precise localization. A recent study by Hur et al. also showed that PET/CT is helpful in both N and M staging and therefore aids in the patient selection for surgery or avoiding unnucessary laparotomy [17]. The guideline also recommends PET for evaluation of therapy response and to make the decision of continuing the ongoing therapy or stopping and redirecting the patient to other salvage therapies. Our study also supports the idea of routine use of PET or preferably PET/CT in the posttherapy follow up.

As a result, when statistical results are reviewed, it is recognized that high Kappa values (>0.7) for local recurrence and local lymph node metastasis were calculated and that this concordance showed PET/CT had a similar diagnostic power to spiral CT. However the low Kappa levels calculated for distant metastasis revealed that there was a significant discordance between two techniques. This situation was mainly due to the sclerotic bone lesions and millimetric lung nodules that PET/CT had failed to show. The clinical significance of this situation is uncertain as the millimetric nodules were proved to be non-malignant in the clinical course. When it comes to skeletal metastases, not sclerotic but lytic lesions can be apparently diagnosed by PET/CT. The mechanism of low FDG uptake in sclerotic lesions have been hypothesized before. FDG scanning depends on the metabolic activity of the tumor. Because sclerotic lesions include a smaller amount of metabolically active cells, they have a lower FDG uptake cannot be easily shown by FDG PET. But lytic metastases can be detected successfully [18]. So the clinical experience we get from this study is that the complementary role of FDG PET/CT in the clinical follow up of gastric cancer patients cannot be ignored, keeping in mind its ability to provide a whole body imaging and much less radiation exposure compared to spiral CT.

## Conclusion

According to the results of this study, we conclude that PET/CT is a complementary imaging method which can be successfully used in both preoperative and posttherapy evaluation of gastric cancer.

## Competing interests

The authors declare that they have no competing interests.

## Authors' contributions

EO participated in the design of the study and drafted the manuscript. MA and CS performed the statistical analysis, documentation of the data, literature analysis and participated in drafting the manuscript. OK conceived of the study, and participated in its design and coordination. All authors read and approved the final manuscript.
